# Comparison of Bone Mineral Density among Alcoholism and Nonalcoholism Athlete and Nonathlete Subjects

**DOI:** 10.5005/jp-journals-10018-1086

**Published:** 2014-01-22

**Authors:** Siros Hosseini, Roholla Valizade, Fateme Daraei Heydarabadi

**Affiliations:** 1 Department of Physical Education and Sport Science, Bebahan Branch, Islamic Azad University, Bebahan, Iran; 2 Sama Technical and Vocational Training College, Omidiyeh Branch, Islamic Azad University, Omidiyeh, Iran; 3 Department of Physical Education and Sport Science, Bebahan Branch, Islamic Azad University, Bebahan, Iran

**Keywords:** Bone mineral density, Alcoholism, Nonalcoholism, Athlete, Nonathlete.

## Abstract

**Background:**

The aim of this study was to compare bone mineral density among alcoholic, nonalcoholic and nonathlete subjects.

**Materials and methods:**

The group consisted of physically active people. A questionnaire was given to both alcoholics and nonalcoholics and 28 persons were randomly selected (15 members who consumed alcohol and 13 who did not). In order to collect the data on mineral aggregation, a testing device (DEXA) was used. The data were analyzed using SPSS software.

**Results:**

It was found that higher bone aggregation in each of the two athletic group in comparison with the nonathletic group (p < 0.05).

**Conclusion:**

Exercise may have a positive impact on bone mineral density.

**Abbreviations:**

BMD: bone mineral density; SPSS: Statistical package for social science.

**How to cite this article:** Hosseini S, Valizade R, Heydarabadi FD. Comparison of Bone Mineral Density among Alcoholism and Nonalcoholism Athlete and Nonathlete Subjects. Euroasian J Hepato-Gastroenterol 2014;4(1):1-3.

## INTRODUCTION

Osteoporosis is one of the most common health problems in the world and this occurs in the form of bone fractures that progresses with ages.^1^ Osteoporosis may induce anomaly in the neurotic system and advances slowly and without any notable symptom.^2^ Therefore, no curative therapy has been found for this clinical problem. Only with exercise, good nutrition and consumption of calcium, the risks of osteoporosis can be reduced.^3-5^ Additional factors related to progression of osteoporosis are age and gender.^6^ Sports have been indicated as one of the most important and effective means in the prevention of osteoporosis.^7^ Exercise not only preserves but also stimulates bone formation while reducing osteoporosis risks such as mineral aggregation. It also strengthens equilibrium of muscles and thereby reduces breakage risks significantly.^8^ Micklesfield et al^9^ have reported that bone bulk aggregation is three times more in people who exercise than in individuals who do not exercise. Lawson et al^10^ have indicated that bone aggregation is found more in girl students than others. Similar results have been seen in soccer players,^11-13^ weight lifters,^14^ and tennis players.^15^ Investigators agree that correct lifestyle is one of the factors necessary for good health and results in accession or prevention of osteoporosis especially for older age groups.^16^ With the exception of sports, other factors also have an important role in lifestyle-related diseases. Investigators have shown negative effects of alcohol consumption. Spencer et al^17^ have reported that alcohol consumption causes a significant bone reduction in people aged between 31 and 47 years. Williams et al^18^ have also indicated that there is an increase in bone aggregation in individuals who have a history of alcohol consumption. Taken together, a panel of national institutes has paid attention and has indicated that alcoholism is one of the causes of osteoporosis. However, these findings need to be optimized by further investigations in different part of the world.^19^

**Fig. 1:**
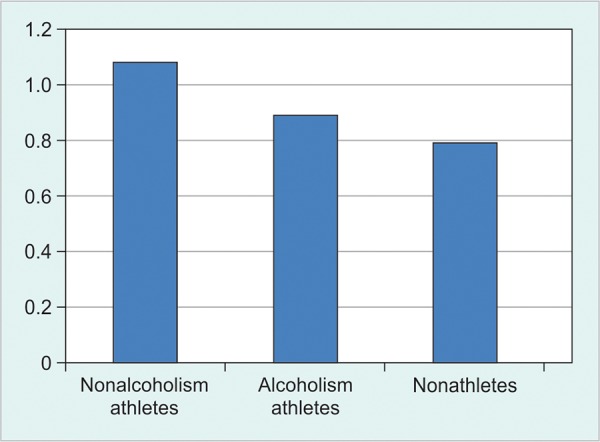
The bone aggregation in each of the three groups is shown (g/cm^2^)

## MATERIALS AND METHODS

The study was conducted on athletic and nonathletic men (aged 45-50 years). The group consisted of physically active people. A questionnaire was given to both alcoholics and nonalcoholics and 28 persons were randomly selected (15 consuming alcohol and the rest 13 with no history of alcohol intake). In order to collect the data on mineral aggregation, a testing device (DEXA) was used along with a questionnaire on the past sport activity of the participants, recording their history of alcohol consumption along with a premedical history of the individual. The American System^20^ considered as one of the most precise and reliable method was used to find the bone mineral aggregation rate in the leg. This test was conducted at the Khozestan osteoporosis testing center by radiology experts. The resulting data were analyzed using used SPSS software.

## RESULTS

The bone aggregation test of the three groups is shown in Figure 1. The results show that the nonalcoholism athlete group without alcohol consumption showed significant higher bone aggregation compared with two other groups (alcoholism athlete and nonathlete subjects). In addition, the Chase-Chace effect or the result of least significant difference test showed that the athletic group who consumed alcohol to have high bone aggregation in proportion to the nonathletic group.

## DISCUSSION

It appears that sports may have caused a mechanical effect that may induce increased mineral aggregation in the bones. Therefore, continued sports activity is of great importance especially in advanced ages. Nevertheless, it was shown that athletics who consumed alcohol had low bone aggregation when compared with athletes of nonalcoholic group. Wosje and Kalk Warf^21^ reported that women and men above the age of 20 who consumed greater amounts alcohol had more bone aggregation. In addition, investigators have also reported increase in the risk of astopeny with the increase in consumption of alcoholic drinks. This study also showed a negative effect of alcohol consumption on bone mineral density.
